# Thermodynamic phases in two-dimensional active matter

**DOI:** 10.1038/s41467-018-07491-5

**Published:** 2018-11-28

**Authors:** Juliane U. Klamser, Sebastian C. Kapfer, Werner Krauth

**Affiliations:** 1 Laboratoire de Physique Statistique, Département de physique de l’ENS, Ecole Normale Supérieure, PSL Research University, Université Paris Diderot, Sorbonne Paris Cité, Sorbonne Universités, UPMC Univ. Paris 06, CNRS, 75005 Paris, France; 2 Max-Planck-Institut für Physik komplexer Systeme, Nöthnitzer Str. 38, 01187 Dresden, Germany; 3 Theoretische Physik 1, FAU Erlangen-Nürnberg, Staudtstr. 7, 91058 Erlangen, Germany; 4 Department of Physics, Graduate School of Science, The University of Tokyo, 7-3-1 Hongo, Bunkyo, Tokyo 113-0033 Japan

## Abstract

Active matter has been much studied for its intriguing properties such as collective motion, motility-induced phase separation and giant fluctuations. However, it has remained unclear how the states of active materials connect with the equilibrium phases. For two-dimensional systems, this is also because the understanding of the liquid, hexatic, and solid equilibrium phases and their phase transitions is recent. Here we show that two-dimensional self-propelled point particles with inverse-power-law repulsions moving with a kinetic Monte Carlo algorithm without alignment interactions preserve all equilibrium phases up to very large activities. Furthermore, at high activity within the liquid phase, a critical point opens up a gas–liquid motility-induced phase separation region. In our model, two-step melting and motility-induced phase separation are thus independent phenomena. We discuss the reasons for these findings to be common to a wide class of two-dimensional active systems.

## Abstract (web summary)

Adapting statistical physics tools to study active systems is challenging due to their non-equilibrium nature. Here the authors use simulations to present a phase diagram of a 2D active system, showing a two-step melting scenario far from equilibrium along with gas-liquid motility-induced phase separation.

## Introduction

Active matter has been intensely studied for its wealth of intriguing properties, such as collective motion^1^, motility-induced phase separation (MIPS)^2^, and giant fluctuations away from criticality^3^. However, the precise connection of the steady-state behaviour of active materials with all phases appearing in the equilibrium phase diagram has remained unclear.

In two spatial dimensions, the analysis of active matter builds upon the very rich equilibrium behaviour of two-dimensional (2D) particle systems, for which the existence of a crystalline phase featuring Bragg peaks and long-range positional order is excluded by the Mermin–Wagner theorem^4^. Nevertheless, two-dimensional particle systems generically undergo two phase transitions. One transition separates the liquid phase (which has short-range positional and orientational order) from a hexatic phase (with short-range positional yet quasi-long-range orientational order). A second transition separates the hexatic phase from a solid (albeit non-crystalline) phase with quasi-long-range positional order and long-range orientational order. The experimental^5–7^ and theoretical^8–11^ understanding of these phases and of the phase transitions by which the dissociated orientational and positional orderings^12^ build up is very recent. Today, the most complete picture is available for the particularly interesting model of particles with inverse-power-law repulsions (*r*^−*n*^, where *r* is the interparticle distance), which leads all the way from the hard-disk model (for *n* → ∞) to the Coulomb gas (for *n* = 1). Because of the absence of an energy scale (e.g. for the Lennard–Jones potential, an energy scale is provided by the depth of the potential minimum), the equilibrium behaviour of the inverse-power-law model depends only on a single parameter, which combines temperature, overall potential strength, and density. In the equilibrium phase diagram of all these models with inverse-power-law interactions, the three phases (liquid, hexatic, solid) are present. In numerical simulations using Monte Carlo methods, the identification of all three thermodynamic phases is far more difficult for the hard-disk model (*n* → ∞) than for softer potentials *n* ~ 6.

In this work, we use an “active” non-equilibrium generalisation of the above equilibrium model as a minimal approach to 2D active materials, where self-propelled particles interact via inverse-power-law repulsions (but without alignment interactions). The dynamics is modelled by a kinetic Monte Carlo (MC) algorithm, which allows us to smoothly access the non-equilibrium state from an equilibrium state, by tuning a single control parameter. We map out the full quantitative phase diagram, and we show that the active system preserves all equilibrium phases. In particular, we positively identify the hexatic phase, even at large activity, from the decay of the orientational and positional correlation functions that we compute to high precision. We thus demonstrate that positional and orientational degrees of freedom continue to be dissociated in the two-dimensional active system. With increasing activity, the transitions between these phases shift to higher densities, and the two-step melting scenario is maintained. At a high enough activity, in the liquid phase, a critical point opens up a gas–liquid MIPS region. This demonstrates that the two-step melting and MIPS are independent phenomena. As our model is minimal, we expect this finding to be robust and the independence to be common to a wide class of two-dimensional active systems.

## Results

### Kinetic Monte Carlo dynamics

**Fig. 1:**
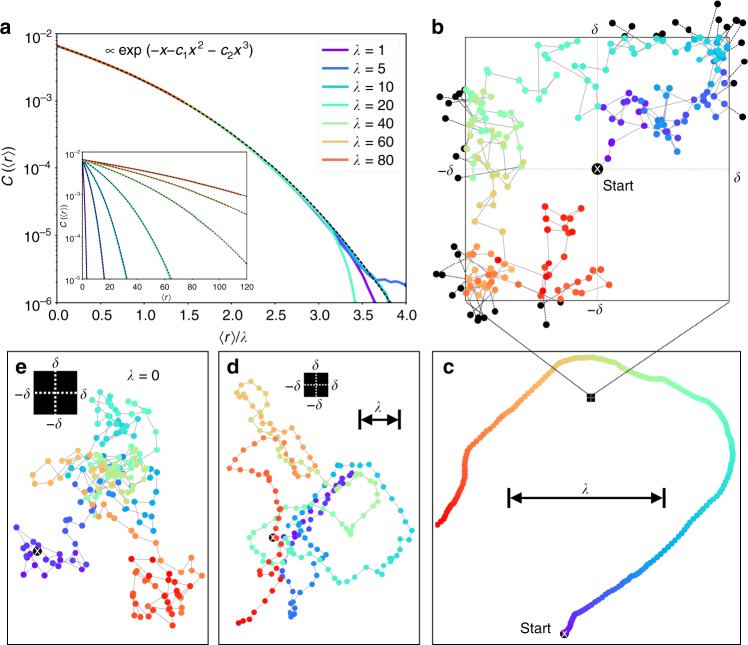
Kinetic Monte Carlo algorithm. **a** Autocorrelation of proposed displacements as a function of the covered distance for a single 2D active particle. The data collapse for widely different activities allows the definition of a persistence length *λ* (see Methods) (see inset for raw data without rescaling). **b** Time evolution of proposed displacements in a box of size [−*δ*, *δ*]^2^. The displacement **є**(*t*) is sampled from a bivariate normal distribution with standard deviation *σ* (here $\sigma \ll \delta$), centred at the previous displacement **є**(*t* − 1). Positions sampled outside the box (black points) are folded back, implementing the reflecting boundary condition (see Methods). **c** Trajectory for a single particle ${\bf{r}}(t) = \mathop {\sum}\nolimits_{k = 1}^t$ **є**(*k*), with the corresponding history of displacements from (**b**). The colour gradient changing with time allows to connect (**b**, **c**), e.g. the last displacements in **b** are in the third quadrant thus the particle in **c** moves to the lower left etc. **c**–**e** Sampled trajectories, illustrating the transition from a passive random walk ($\sigma \gg \delta$ in **e**) to a persistent random walk ($\sigma \ll \delta$ in **c**)

The kinetic discrete-time MC dynamics for active matter, that we use, is closely related to the active Ornstein–Uhlenbeck process^13^. The proposed displacements of a single particle are correlated in time, leading to ballistic local motion characterised by a persistence length *λ* which measures the degree of activity (Fig. 1). The correlations decay exponentially, so that the long-time dynamics remains diffusive. Detailed balance is satisfied only for vanishing activity, that is, in the limit *λ* → 0. Cooperative effects between multiple active particles are introduced through a repulsive inverse-power-law pair potential. A 1/*r*^6^ potential is chosen. (This is justified later.) Individual displacements of particles are accepted or rejected with the standard Metropolis criterion. The rejections are without incidence on the sequence of proposed moves, which are also uncorrelated between particles so that the active many-particle system is without alignment interactions.We perform simulations of *N* particles in a rectangular box of volume *V* at constant density *ϕ* and at constant persistence length *λ*. (For definitions see Methods.)

### Two-step melting of active system and its equilibrium limit

**Fig. 2:**
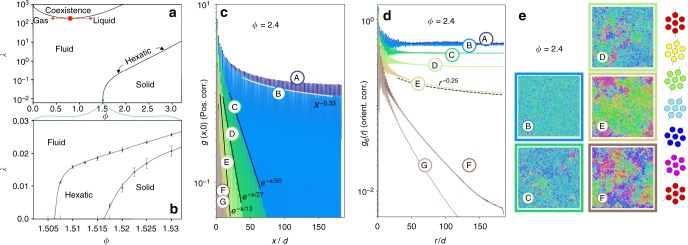
Full phase diagram and two-step melting. Depicted results are for *N* ~ 4.4 × 10^4^ particles and *δ* = 0.1. **a** Activity *λ* vs. density *ϕ* phase diagram. MIPS between a liquid and a gas, at high *λ*, is situated far above the solid–hexatic–liquid melting lines. The red dot indicates a possible critical point. **b** Two-step melting for small *λ* with shift of transition densities to higher values with increasing *λ*, preserving the equilibrium phases. Two-step melting from the solid is induced by density reduction (as in equilibrium) but also by an increase in *λ*. (See Methods for definition of error bars.) **c**–**e** Activity-induced two-step melting high above the equilibrium melting densities (*ϕ* = 2.4, A: *λ* = 0.987; B: *λ* = 0.999; C: *λ* = 1.018; D: *λ* = 1.033; E: *λ* = 1.045; F: *λ* = 1.064; G: *λ* = 1.079). **c** Positional correlation function *g*(*x*,*y*) along the *x* axis, in units of the global mean interparticle distance *d* = (*πN*/*V*)^−1/2^. **d** Orientational correlation function *g*_6_(*r*). **e** Snapshots of configurations, particles colour-coded with their local orientation parameter *ψ*_6_. A and B are quasi-long-ranged in *g*(*x*, 0) and long-ranged in *g*_6_(*r*), thus corresponding to the solid phase. C, D and E are short-ranged in *g*(*x*, 0) and quasi-long-ranged in *g*_6_(*r*), thus corresponding to the hexatic phase. F and G decay exponentially in both correlation functions and thus correspond to a liquid. (For definitions see Methods)

At all densities and activities we observe a unique non-equilibrium steady state in which spatial correlation functions are well-defined. At vanishing activity *λ*, where the particle dynamics is passive, we recover the equilibrium phase diagram with the established two-step melting scenario (see Fig. 2). In particular, we observe the exponential decay of positional and orientational correlation functions (no order) in the liquid, the power-law decay of orientational correlations (quasi-long-range order) yet exponential decay of positional correlations in the hexatic phase, and long-range orientational correlations together with positional power-law decay in the solid. Our choice of the 1/*r*^6^ interaction potential is particularly amenable to simulation because of its “soft” hexatic phase, characterised by small positional correlation lengths^11^. Soft hexatics feature much shorter MC correlation times and reach the thermodynamic limit for smaller system sizes than the hard-disk-like hexatics^10^ (that correspond to a 1/*r*^*n*^ interaction with *n* → ∞).

In 2D equilibrium systems, a true crystal with Bragg peaks and long-range positional order exists only in the *ϕ* → ∞ limit, as a consequence of the Mermin–Wagner theorem^4^. However, at finite density there is a solid phase where the positional order is quasi-long-ranged. We find that the solid phase of the active system also exhibits algebraic positional order, just as in equilibrium. Decreasing the density or, remarkably, increasing the activity weakens the positional correlations and eventually melts the solid (Fig. 2a, b). Close to the melting transition, the algebraic decay of the positional correlations can be clearly observed in our simulation data (points A and B in Fig. 2c). Together with the long-range orientational order, this explicitly identifies the solid phase (Fig. 2d).

The hexatic phase in equilibrium is characterised through a lower degree of order than the solid, namely through short-range positional correlations and algebraic orientational correlations. We find precisely such a phase in the active system, in a narrow strip of densities below and activities above the solid phase (see Fig. 2a, b). Starting from the solid, we indeed observe positional correlations that change qualitatively upon a minute increase in activity while leaving the orientational correlations almost unchanged, leading to hexatic order (from point B to point C in Fig. 2c). Positional correlations in the hexatic phase decay exponentially beyond the correlation length but the orientational correlations remain quasi-long-ranged (points C through E in Fig. 2d, e). On moving towards the liquid at any point within the hexatic phase, the positional correlation lengths decrease, resulting in a strikingly soft hexatic close to the liquid–hexatic transition (point E in Fig. 2c–e). This soft hexatic maintains orientational quasi-order with extremely short-ranged positional correlations, even at densities for which the equilibrium system is already deep inside the solid phase. Increasing the activity thus shifts the equilibrium phase boundaries towards higher densities. The stability of the partially ordered hexatic phase is remarkable especially as it takes place for persistence lengths *λ* significantly larger than the global mean interparticle distance *d* = (*πN*/*V*)^−1/2^. Our massive computations give no indications of a direct transition from the solid into the liquid state, even at the highest densities.

### Motility-induced phase separation as liquid–gas coexistence

**Fig. 3:**
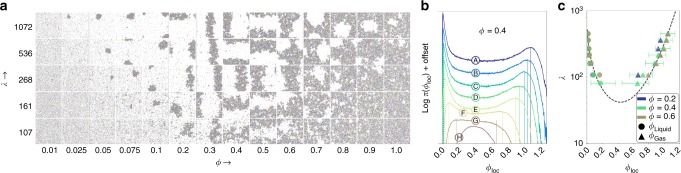
Characterisation of motility-induced phase separation. Data for *N* ≈ 1.1 × 10^4^, *δ* = 0.7. **a** Snapshots of configurations close to the onset of liquid–gas coexistence. (Particles represented in arbitrary size and colour-coded, as in Fig. 2e, according to their local orientation parameter.) U-shaped phase boundary is apparent. Orientational order is short-ranged in both liquid and gas phase, thus the coloured snapshots appear grey. At constant activity *λ*, the liquid volume fraction grows with increasing density until the liquid entirely fills the system. The location of the critical point depends on *δ* and it appears at a smaller density and *λ* than in Fig. 2. **b** Histograms of local densities (see Methods for definition) for a variation of activities at constant *ϕ* = 0.4 (A: *λ* = 450; B: *λ* = 359; C: *λ* = 268; D: *λ* = 214; E: *λ* = 161; F:*λ* = 107; G: *λ* = 80; H: *λ* = 54; For better presentation, histograms are shifted along the *y *axis with increasing *λ*). Transition from a single-peaked to a double-peaked distribution and increasing separation of peaks with increasing *λ*. **c** Densities of liquid and gas (identified through peak position as in **b**) in an activity *λ* vs. local density *ϕ*_loc_ diagram. This demonstrates independence of phase densities on global density for fixed *λ* and validates the phase-separation picture. For a clear presentation, we plot exemplifying error bars for *ϕ* = 0.4 only, which were obtained from the width of the corresponding peak in **b**

MIPS^2^ has been frequently reported in 2D active systems but agreement on the nature of the coexisting phases was not reached^14–17^. Recent work^14^ in an active dumbbell system proposed that MIPS continuously extends from the equilibrium liquid–hexatic transition region and that one of the separated phases preserves some degree of order. This is not the case in our system: We clearly observe MIPS as a U-shaped region of liquid–gas coexistence (Fig. 3a) with an onset at high activity and at relatively low density. Both competing phases in the phase-separated state feature exponential decay of orientational and positional correlation functions. They are thus both disordered (see the configuration snapshots in Fig. 3a that are colour-coded by local orientational order). MIPS is therefore clearly separated from the melting (Fig. 2a). During an MC simulation, a coarsening process generally precedes macroscopic phase separation in the time evolution towards the steady state. In the active 1/*r*^6^ system, the transient coarsening leading up to MIPS is likely to be overcome at earlier times than for hard disks. This makes it easier for us to distinguish MIPS from the formation of a steady-state gel^18^, although we do not expect the nature of the coexisting phases participating in MIPS to depend on the softness of the potential.

### Further characterisation of MIPS

**Fig. 4:**
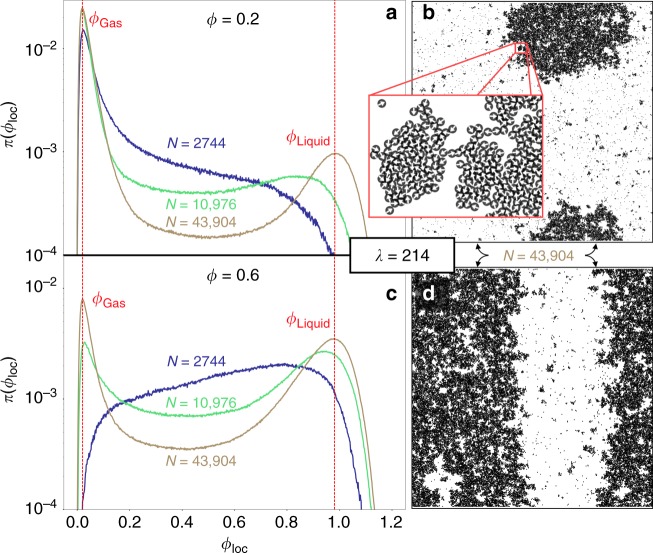
Finite-size scaling of local density histograms. Data with *λ* = 214 and *δ* = 0.7. **a**, **c** Local density histograms (see Methods for definitions) for global densities *ϕ* = 0.2 and *ϕ* = 0.6 (identical *x* axis used). Local density peaks sharpen with increasing system size *N*, and are located at the same value of *ϕ*_loc_, demonstrating that in the MIPS region gas and liquid densities are independent of the global density. **b**, **d** Snapshots of configurations at global densities corresponding to **a**, where the liquid is the minority phase, and **c**, where the liquid is the majority phase by volume fraction (cf. height of the peaks in **a** and **c**). Inset in **b** Direction of motion of the individual particles indicated by arrows, illustrating the origin of MIPS. The directions of motion are uncorrelated inside the homogeneous liquid and gas. Only particles at the interface move towards the interior of the liquid patch, enclosing particles of the high-density region

The analogy with the liquid–gas coexistence in equilibrium simple fluids suggests the interpretation of the onset of MIPS as a critical point. Indeed, below the onset, the system remains homogeneous at large length scales with a single-peaked local density distribution (see point H in Fig. 3b). Above the onset of MIPS, the local densities develop a bimodal distribution. The peak positions separate further as *λ* increases, quantifying the above-mentioned U-shape. Moreover, at a given *λ*, the peak local densities in the coexistence region are independent of the global density *ϕ* (Fig. 3c). This is further substantiated by a finite-size scaling analysis at constant *λ* (Fig. 4). The phenomenology thus agrees with that of an equilibrium phase coexistence where the relative proportions of the liquid and gas adapt to the global density, but where the degree of order of each of the phases and their densities remain unchanged. Inside the coexistence region, at small densities, we observe an approximately circular bubble of liquid inside the gas, followed by a stripe-shaped form that winds around the periodic simulation box, and then followed by a bubble of gas inside the liquid. In finite (*N*, *V*, *T*) equilibrium systems, this complex behaviour is brought about by the interface free energy^19,20^ which vanishes at the critical point. A detailed analysis of the homogeneous phases, but also of the phase-separated systems, reveals the origin of the phase separation in the kinetic MC model. In the bulk of the coexisting liquid and gas phases, the directions of motion of individual particles are uncorrelated beyond a very small length scale (Fig. 4b). At the liquid–gas interface, however, a majority of the increment vectors point inwards towards the liquid phase. Even though a theoretical framework as reliable as statistical mechanics is currently lacking, the effective cohesion in non-equilibrium is often attributed to the so-called swim pressure due to active motion^21–23^.

## Discussion

In this work, we have first demonstrated, by numerical simulation for the considered model of active-particle systems, that the two-step melting from a solid to a hexatic to a liquid phase is preserved away from equilibrium (where the scenario is well established) even up to quite high activities. There is reason to believe that in the limit of small activities, active-particle systems can retain an effective equilibrium description at a coarse-grained level^13,23–26^. Our work considerably extends this finding, as we positively identify the continuation of the equilibrium phases at large activities. In our opinion, the stability of the hexatic phase (which we explicitly identify in this paper through the orientational and positional correlation functions) is particularly noteworthy. Its presence illustrates that the dissociation of the ordering between the orientational and positional degrees of freedom is preserved even at large activities. We conjecture that for all activities, the hexatic phase is stable in a finite density interval and that, in the limit of infinite activities, the liquid–hexatic phase transition shifts to infinite densities.

Most importantly, our analysis conclusively identifies the hexatic steady state through the defining exponential decay of its positional correlation functions and algebraic decay of orientational correlation functions. Even though the existence of the hexatic far from equilibrium was suggested earlier in driven granular experiments^27^, the correlation functions could not be evaluated precisely in these experiments, because the system sizes were very small.

Second, in this work, we have given unambiguous evidence that MIPS in our 2D active materials manifests itself as a liquid–gas coexistence, that is, with phases that connect to equilibrium phases, and that feature short-range order. This thus excludes crystals, polycrystals, and gel phases. In our simulations, the infinite-time steady state is effectively reached on the available run-time scales because the hexatic phase in the 1/*r*^6^ potential is considerably softer than, for example, in hard-disk systems^11^. Moreover, the MIPS state is clearly observed, whereas for hard disks, the dynamics appears sluggish and gel-like^18^, although we do not expect this observation to reflect the steady state. Even though the 1/*r*^6^ model could be relatively remote from the experimental colloidal particles, it serves our purpose to describe the steady state of the active-particle system in the *t* → ∞ limit. In the classic hard-disk melting problem, the access to the steady state (for passive systems: the thermodynamic equilibrium) can be very time-consuming. This bottleneck has prevented a conclusive resolution for decades. In hard disks, the steady state was first found using specialised algorithms^10^ which cannot be employed for active systems. The firm understanding of systems with relatively weak potentials will in our opinion open the way for the study of systems with stiffer potentials. It appears possible that the reduction in the number of parameters that is proper to the inverse-power-law potential also reduces the number of effective parameters in the active system. The entire phase behaviour could then be described through few parameters, in essence through a suitably scaled interaction strength and activity.

The hexatic phase and the MIPS are separated by the homogeneous liquid phase, which is both the high-density end of MIPS and the low-density end of the order–disorder transitions. Intriguingly, the subtle hexatic phase survives even at considerably high activities. Our massive computations allow us to reach the steady state even for strong activities and for high densities. However, support for our conjecture of the stability of the separating liquid phase and of the hexatic state in the *λ*, *ϕ* → ∞ limit, would have to rely on deeper theoretical understanding than is today available. This relates to the question of how the KTHNY theory of 2D melting^8,28,29^, built for equilibrium systems, would extend to active systems. We find that the limiting exponents (0.33 and 0.25) for the positional and orientational correlations predicted in equilibrium by the KTHNY theory seem to be robust even at high activity (e.g. Fig. 2c, d). This points towards the possibility that a coarse-grained free-energy functional exists^22,24,25,30^, such that both MIPS and the two-step melting are covered under one effective equilibrium theory. Derivatives of the free energy would allow for the rigorous definition of state variables, such as pressure. Although pressure is not a state variable in generic active systems, our model belongs to the narrow class with torque-free dynamics where pressure could be defined^21,22^. Computing pressure in our model is not straightforward and is left for future work. The effect of activity could be reflected as a change in the effective pressure. For example in Fig. 2a, at sufficiently high activity, an increase of density induces a gas–liquid coexistence, in striking analogy with the van-der-Waals physics found in equilibrium fluids of attractive particles (e.g. the Lennard–Jones system). Comparing with the phase diagram of Fig. 2a would suggest that the effective pressure decreases with increasing activity. An even further density increase finally induces the ordering transitions, which again resembles the behaviour of an equilibrium Lennard–Jones system. This only strengthens the analogy with an equilibrium scenario.

In equilibrium, the nature of the two-step melting phase transitions depends on the softness of the particles, which can be tuned via the exponent *n* of the inverse-power-law pair potential *U*(*r*) ∝ 1/*r*^*n*^. The two-step melting scenario for very soft particles (*n* ≲ 6) comprises two continuous transitions, whereas for harder particles with *n* ≳ 6 the liquid–hexatic transition becomes first order^11^. One may speculate that the activity plays a similar role as the hardness of the particles and that at high activities the liquid–hexatic transition becomes first order.

Although MIPS^2^ has been frequently reported in active systems, there are conflicting results on the precise nature of the coexisting phases^14–17^. For example, it was reported as a coexistence between a “solid-like and gas state”^16^, referred to as a coexistence between a “dense large cluster and a dilute gas phase”^17^, diagnosed as the coexistence between a liquid state and a polycrystal, with domains of different orientational order^14^, or referred to a coexistence between a “dense and dilute phase”, where the dense phase “exhibits structural properties consistent with a 2D colloidal crystal near the crystal–hexatic transition point”^15^. In addition to being specific to a particular physical system, previous analyses of MIPS phases may also have suffered from insufficient system sizes and warm-up times such that the large-system steady states were often not attained (see Methods for details on our checks of convergence from structurally different initial configurations).

Earlier models for active matter showing MIPS were primarily based on Brownian and molecular dynamics simulations^15,16,24,31,32^. We show that MIPS can be reproduced within kinetic MC dynamics, without added equilibrium-like mixing steps as in ref.^18^ Indeed, the direction of the individual persistent particle motion suffices to produce the effect: Particles may be kinetically arrested for persistence lengths larger than the mean free path, leading to density inhomogeneities, where particles in denser parts of the system are walled in by particles at the interface. However, this does not unhinge the coarsening mechanism: The size of homogeneous domains increases with time, and the infinite-time steady state in a finite system is characterised by only two coexisting domains so that, for the studied 1/*r*^6^ potential, a gel phase is clearly absent. MIPS appears naturally in the kinetic MC approach, and we support the idea that it is a generic feature of active matter in 2D. Our inverse-power-law interactions provide a tunable set of active-matter models to study phase transitions and phase coexistence. A particular pressing question concerns the stability of the two-step melting scenario, especially of the hexatic phase, under the influence of additional alignment interactions^33,34^. We expect a modified version of our kinetic MC approach to permit access to large enough systems for this question to be resolved.

## Methods

### Kinetic MC dynamics for active particles

We use a modified Metropolis algorithm that breaks detailed balance. In each Monte Carlo step, a displacement by an amount **є**_*i*_ is proposed for a single randomly chosen particle *i*. The move is accepted with the Metropolis probability *P* = min[1, *e*^−*β*Δ*E*^], where Δ*E* is the change in the total energy *E* caused by the displacement and *β* = (*k*_B_*T*)^−1^ is the inverse temperature in the passive dynamics. The total energy is $\mathop {\sum}\nolimits_{i < j} {\kern 1pt} U\left( {{\bf{r}}_i - {\bf{r}}_j} \right)$, where we choose the pair-potential *U*(*r*) = *u*_0_ × (*γ*/*r*)^6^, with *γ* playing the role of the particle diameter. Writing *βU*(*r*) = Γ × (*d*/*r*)^6^ with the global mean interparticle distance $d = \sqrt {V{\mathrm{/}}(\pi N)}$, dimensionless interaction strength Γ = *βu*_0_ × (*πϕ*)^3^ and dimensionless density *ϕ* = *γ*^2^*N*/*V*, it can be easily seen that Γ plays the role of an effective reciprocal temperature that scales as *ϕ*^3^ in equilibrium. Without losing generality, we fix the energy and length scales in our system to be *βu*_0_ = 1 and *γ* = 1, leaving only *ϕ* as a parameter. Our simulations are performed in a $\left( {7:4\sqrt 3 } \right)$ simulation box with periodic boundary conditions and the soft-sphere potential is truncated as $\tilde U(r) = U\left( {{\mathrm{min}}\left( {r,1.8} \right)} \right)$^11^.

We introduce activity into the dynamics by choosing the proposed displacement **є**′_*i*_ of particle *i* based on the previously proposed displacement **є**_*i*_ of the same particle. The correlation is introduced in two stages. First, a random vector **η** is sampled from a bivariate normal distribution ∝exp[−(**η** − **є**_*i*_)^2^/2*σ*^2^], where *σ* is the standard deviation. In the second stage, **є**′_*i*_ is generated from **η** using the folding scheme

1 $${\epsilon _{i,z}^\prime \to} \left\{\begin{array}{*{20}{l}}q_{i,z} - \delta & {\mathrm{if}}\,q_{i,z} < 2\delta \\ 3\delta - q_{i,z} & \hskip 9pt {\mathrm{if}}\,q_{i,z} \ge 2\delta ,\end{array}\right.$$

with *z* ∈ {x, y} and *q*_*i*,*z*_ = (*η*_*z*_ + *δ*) mod 4*δ*, with $a\,{\mathrm{mod}}\,b = a - b\left\lfloor {{\textstyle{a \over b}}} \right\rfloor$, i. e., 0 ≤ *q*_*i*,*z*_ < 4*δ*, where $\left\lfloor a \right\rfloor$ is the floor function. The folding scheme limits the size of the proposed displacements and keeps the dynamics local. The folding scheme is equivalent to a random walk of the displacement variables **є**_*i*_ in a [−*δ*, *δ*]^2^ box with reflecting boundary conditions, see Fig. 1b. Note that the random walk of the displacements is independent of the positions of the particles, as the new increment persists whether the displacement was accepted or not.

The square-shaped displacement box introduces a small degree of anisotropy into the dynamics for *λ* > 0. However, we explicitly verify that the resulting steady state is unaffected with respect to the properties concerning this letter. At small densities in the gaseous state, where *λ* is much smaller than the mean free path, the kinetic MC dynamics effectively reverts to detailed-balance dynamics as interactions between particles are rare. At higher densities, all large proposed displacements have vanishing Metropolis probabilities, thus leading to effectively isotropic dynamics. In our numerical observation anisotropic effects are undetectable within other sources of noise.

### Probability of increments and persistence length

In continuous time, the increment variable **є** evolves according to a diffusion equation *∂*_*t*_*P*(**є**, *t*) = ${\textstyle{{\sigma ^2} \over 2}}$Δ_**є**_*P*(**є**, *t*), with vanishing probability flux through the boundary of the displacement box. The steady-state distribution in the infinite-time limit is the uniform distribution *P* = (2*δ*)^−2^. By a Fourier ansatz, one readily finds that the autocorrelation time *τ* of increments is dominated by the first harmonic of the displacement box, and that for large *t*, the autocorrelation function decays as

2 $$C(t) \equiv \left\langle {{{\epsilon }}\left( {t_0 + t} \right) \cdot {{\epsilon }}\left( {t_0} \right)} \right\rangle \propto {\mathrm{e}}^{ - t/\tau },{\mathrm{where}}\,\tau = \frac{{8\delta ^2}}{{\pi ^2\sigma ^2}}.$$

The position of a free particle evolves as ${\bf{r}}(t) \equiv {\bf{r}}\left( {t_0} \right) + {\int}_{t_0}^t {\kern 1pt} {\kern 1pt} {\mathrm{d}}s$ **є**(*s*). For times shorter than the autocorrelation time, *t* ≲ *τ*, its mean displacement is essentially given by the increment at time *t*_0_,

3 $$\left\langle {\left| {{\bf{r}}\left( {t_0 + t} \right) - {\bf{r}}\left( {t_0} \right)} \right|} \right\rangle = v{\kern 1pt} t + {\cal O}\left( {\sqrt t } \right),$$

where the drift velocity *v* ≡ |**є**(*t*_0_)| is given by the initial condition of the increment, and the subleading term contains contributions by diffusion of **є**, including reflections, in the displacement box. Averaging over all initial conditions **є**(*t*_0_) with the steady-state uniform distribution, we obtain the mean drift velocity

4 $$\overline v = \frac{\delta }{3}\left[ {\sqrt 2 + {\mathrm{arsinh}}(1)} \right].$$

From Eqs. (2) and (3), we may identify the persistence length $\lambda \equiv \tau {\kern 1pt} \overline v \simeq 0.62\delta ^3\sigma ^{ - 2}$, which provides a length scale separating ballistic motion from diffusive motion. The persistence length defined in this way allows to collapse data for the increment autocorrelations $C\left( {\left\langle r \right\rangle = \lambda x} \right) \propto {\mathrm{e}}^{ - \left( {x + c_1x^2 + c_2x^3} \right)}$ at widely different activities (Fig. 1a). The prefactors of the superexponential terms *c*_1_,*c*_2_ are obtained from a numerical fit.

### Measurements

The orientational order is quantified by the correlation function $g_6(r)$ ∝ $\left\langle {\mathop {\sum}\nolimits_{i,j}^N {\kern 1pt} \psi _6^ \ast \left( {{\bf{r}}_i} \right)\psi _6\left( {{\bf{r}}_j} \right)\delta \left( {r - r_{ij}} \right)} \right\rangle$ of the complex bond-orientational order parameter *ψ*_6_(**r**_*i*_) calculated with Voronoi weights^35^. *g*_6_(*r*) is a measure of the correlation of the local sixfold orientational order at distance *r*. Positional order is studied with the direction-dependent pair correlation function *g*(*x*, *y*). Before averaging this two-dimensional histogram over configurations, each configuration is realigned^10^ such that the Δ*x* axis points in the direction of the global orientation parameter ${\mathrm{\Psi }}_6 = \mathop {\sum}\nolimits_i^N {\kern 1pt} \psi _6\left( {{\bf{r}}_i} \right)$.

**Fig. 5:**
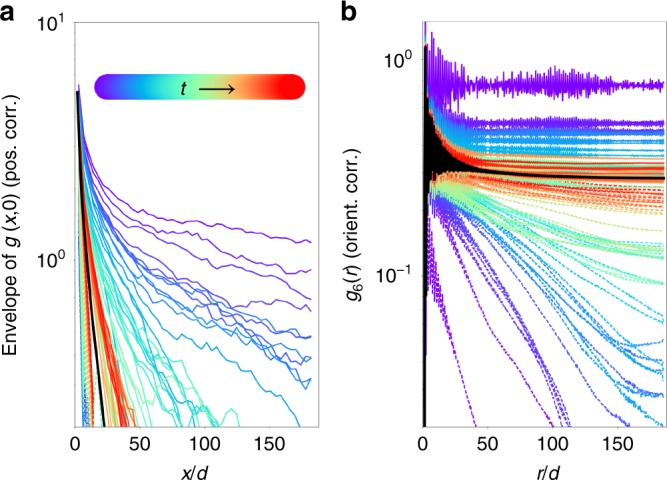
Convergence from two different initial conditions as a function of run-time *t*. Data at *ϕ* = 2.4 with *λ* = 1.0333 and *δ* = 0.1. **a** Positional correlation function *g*(*x*,*y*) along the *x* axis, in units of the global mean interparticle distance *d*. **b** Orientational correlation function *g*_6_(*r*). Both panels show the evolution with run-time *t* (see colour code for run-time ordering in **a**) starting from a random initial configuration (dashed lines) and from an initial configuration with particles arranged in a perfect hexagonal lattice (solid lines). Both initial configurations approach the same hexatic steady state (black solid line)

The data in Fig. 2 were obtained from systems of ~4.4 × 10^4^ particles. Configurations were recorded in time intervals of 2.7 × 10^4^ MC sweeps, with each sweep containing *N* MC steps. Correlation functions in Fig. 2c and d at *ϕ* = 2.4 are ensemble-averaged over 200 configurations, recorded after a warm-up time of 8.2 × 10^6^ MC sweeps. The black solid curves in Fig. 5 show the result for one of the hexatic state points. To ensure that the steady state is reached, we start time evolutions from two structurally different initial particle configurations, one is a random distribution, and the other has particles arranged on a perfect hexagonal lattice (Fig. 5), obtaining equivalent results for positional and orientational correlation functions. This clearly establishes the existence of the hexatic phase in the active system. The melting lines in Fig. 2a and b were obtained from configurations recorded after a warm-up time of at least 5.5 × 10^6^ MC sweeps. The error bars were obtained from the behaviour of 10 short-time ensemble-averages, with each average containing 20 configurations. The lower-activity error bar of the solid–hexatic transition is determined by a state point with all short-time averages of *g*(*x*, 0) having a clear power-law dependence. The high-activity error bar is determined by state points with short-time averages decaying exponentially. The same criterion applied to *g*_6_(*r*) was used for the error bars of the liquid–hexatic transitions.

MIPS is quantified by histograms of local densities, which we compute by covering the system with randomly placed test circles of radius 7.5. The local dimensionless density of each test circle is *ϕ*_loc_ = *γ*^2^*N*_loc_/*V*_loc_, where *N*_loc_ are the number of particle centres located within a circle of area *V*_loc_. The detailed analysis (Figs. 3 and 4) of MIPS uses *δ* = 0.7. The larger *δ* shifts the liquid–gas coexistence phase boundaries, in particular the critical point, to smaller densities and persistence lengths, which drastically shortens the time to reach the steady state. Configuration snapshots in Fig. 3a were taken after 1.1 × 10^7^ MC sweeps. Data in Fig. 3b consist of ensemble-averages over 100 configurations recorded in time intervals of 3 × 10^4^ sweeps. The *N* ~ 4.4 × 10^4^ (*N* ~ 1.1 × 10^4^, *N* ~ 2.7 × 10^3^) data in Fig. 4 were recorded after a warm-up of 1.2 × 10^7^ (2.2 × 10^7^, 1.4 × 10^7^) sweeps. The average consists of 400 (500, 500) configurations recorded in time intervals of 1.09 × 10^4^ (8.7 × 10^3^, 1.7 × 10^4^) sweeps.

## Data Availability

All data and code are available on request to the corresponding author.
